# Retraction: Smart high-yield tomato cultivation: precision irrigation system using the Internet of Things

**DOI:** 10.3389/fpls.2025.1726547

**Published:** 2025-10-28

**Authors:** Frontiers Editorial Office

The journal retracts the 2023 article cited above.

Following publication, concerns were raised regarding the integrity of the images in the published figures. The authors failed to provide a satisfactory explanation during the investigation, which was conducted in accordance with Frontiers’ policies. This retraction was approved by the Chief Executive Editor of Frontiers. The authors received a communication regarding the retraction and had a chance to respond. This communication is recorded by the publisher.

